# Permissive fluid volume in adult patients undergoing extracorporeal membrane oxygenation treatment

**DOI:** 10.1186/s13054-018-2211-x

**Published:** 2018-10-27

**Authors:** Hyoungnae Kim, Jin Hyuk Paek, Joo Han Song, Hajeong Lee, Jong Hyun Jhee, Seohyun Park, Hae-Ryong Yun, Youn Kyung Kee, Seung Hyeok Han, Tae-Hyun Yoo, Shin-Wook Kang, Sejoong Kim, Jung Tak Park

**Affiliations:** 1 Division of Nephrology, Soonchunhyang University Seoul Hospital, Seoul, Republic of Korea; 2 Department of Internal Medicine, Institute of Kidney Disease Research, Yonsei University College of Medicine, 50-1 Yonsei-ro, Seodaemun-Gu, Seoul, 03722 Republic of Korea; 3 Department of Internal Medicine, Seoul National University Bundang Hospital, 82, Gumi-ro 173 Beon-gil, Bundang-gu, Seongnam, Gyeonggi-do 13620 Republic of Korea; 4 Department of Internal Medicine, Institute of Chest Disease, Yonsei University, Seoul, Republic of Korea; 5 Department of Internal Medicine, Seoul National University College of Medicine, Seoul, Republic of Korea; 6 Division of Nephrology and Hypertension, Department of Internal Medicine, Inha University College of Medicine, Incheon, Republic of Korea; 7 Department of Internal Medicine, Hangang Sacred Heart Hospital, Hallym University College of Medicine, Seoul, Republic of Korea

**Keywords:** Acute respiratory distress syndrome, Cardiogenic shock, Extracorporeal membrane oxygenation, Fluid balance, Mortality

## Abstract

**Background:**

Extracorporeal membrane oxygenation (ECMO) is a cardiorespiratory support technique for patients with circulatory or pulmonary failure. Frequently, large-volume fluid resuscitation is needed to ensure sufficient extracorporeal blood flow in patients initiating ECMO. However, excessive overhydration is known to increase mortality in critically ill patients. Therefore, in order to define a tolerant volume range in patients undergoing ECMO treatment, the association between cumulative fluid balance (CFB) and outcome was evaluated in patients undergoing ECMO.

**Methods:**

This retrospective multicenter cohort study was conducted with 723 patients who underwent ECMO in three tertiary care hospitals between 2005 and 2016. CFB was calculated as total fluid input minus total fluid output during the first 3 days from ECMO initiation. The patients were divided into groups that initiated ECMO owing to cardiovascular disease (CVD)-related or non-cardiovascular disease (non-CVD)-related causes. The primary endpoint was mortality within 90 days after ECMO commencement.

**Results:**

Totals of 406 and 317 patients were included in the CVD and non-CVD groups, respectively. In the CVD group, the mean age was 58.4 ± 17.7 years, and 68.2% were male. The mean age was 55.7 ± 15.7 years, and 65.3% were male in the non-CVD group. The median CFB values were 64.7 and 53.5 ml/kg in the CVD and non-CVD groups, respectively. Multivariable analysis using Cox proportional hazards models revealed a significantly increased risk of 90-day mortality in patients with higher CFB values in both the CVD and non-CVD groups. However, the risks were elevated only in the two CFB quartile groups with the largest CFB amounts. Cubic spline models showed that mortality risk began to increase significantly when CFB was 82.3 ml/kg in the CVD group. In patients with respiratory diseases, the mortality risk increase was significant for those with CFB levels above 189.6 ml/kg.

**Conclusions:**

Mortality risk did not increase until a certain level of fluid overload was reached in patients undergoing ECMO. Adequate fluid resuscitation is critical to improving outcomes in these patients.

**Electronic supplementary material:**

The online version of this article (10.1186/s13054-018-2211-x) contains supplementary material, which is available to authorized users.

## Background

Extracorporeal membrane oxygenation (ECMO) is a salvage therapy for patients with severe respiratory and heart failure [1–4]. Patients frequently require large-volume fluid resuscitation during the initial phases of ECMO treatment in order to maintain a sufficient amount of vascular blood drainage for extracorporeal blood flow [5–7]. This need for liberal fluid infusion during ECMO treatment is further exacerbated by the fact that most patients undergoing ECMO treatment are in an intravascular hypovolemic state aggravated by systemic capillary leakage [8, 9]. In addition to the inevitable large-volume fluid resuscitation, administration of blood products for bleeding events accompanying ECMO implantation and reduced urine volume caused by concomitant acute kidney injury (AKI) also play a part in the aggravation of fluid overload in patients undergoing ECMO [10–14]. Furthermore, excessive positive fluid balance during intensive care unit (ICU) stay is reported to affect outcome, and a positive fluid balance was found to increase the risk of mortality in patients with septic shock [15–17]. In addition, large-volume intravenous fluid therapy was associated with more cardiac arrest events and increased pulmonary edema in patients resuscitated from cardiac arrest events [18], and excessive volume overload was found to be linked to poor survival, even in patients undergoing ECMO therapy [19, 20]. Therefore, although large-volume resuscitation is necessary in most patients initiating ECMO, excessive volume overload may lead to poor outcomes. The clinically significant volume overload threshold has not yet been established.

Cumulative fluid balance (CFB) has been widely used as a surrogate marker of fluid therapy during ICU treatment. Higher CFB values after ICU admission were found to increase the risk of AKI development [21]. In addition, CFB has been reported to be associated with worse clinical outcomes in patients with sepsis [15, 22], acute lung injury [23, 24], and cancer [25]. Therefore, the association between CFB during the initial phase of ECMO treatment and outcome was evaluated, and a clinically significant threshold level of CFB that affects outcome was investigated.

## Methods

### Patient selection

**Fig. 1:**
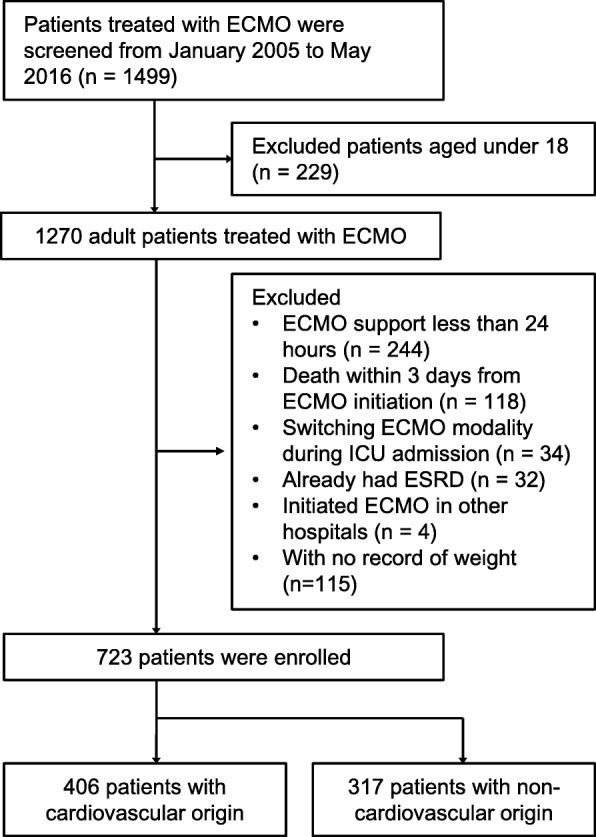
Flowchart of participant enrollment. *ECMO* Extracorporeal membrane oxygenation, *ESRD* End-stage renal disease

Patients who received ECMO at Yonsei University Health System, Seoul National University Hospital, and Seoul National University Bundang Hospital from January 2005 to May 2016 were initially screened (*n* = 1499). Patients who met the following criteria were excluded (*n* = 776): (1) age under 18 years, (2) ECMO support for less than 24 h, (3) death within 3 days from ECMO initiation, (4) switching of ECMO modality, (5) end-stage renal disease with dialysis at admission, (6) transfer after initiating ECMO in other hospitals, and (7) missing data. As a result, 406 patients who underwent ECMO for cardiovascular causes (cardiovascular disease [CVD] group) and 317 patients who underwent ECMO for non-cardiovascular causes (non-CVD group) were included in the final analysis (Fig. 1). Although myocarditis is caused by infection, patients with myocarditis were included in the CVD group on the basis of pathophysiologic considerations. The study protocol was approved by the institutional review boards of Yonsei University Health System and Seoul National University Bundang Hospital. Informed consent was waived by the institutional review boards owing to the retrospective study design.

### Data collection

The patients’ demographic and laboratory data at the time of ECMO initiation were retrieved from electronic medical records. The data included age, sex, height, weight, underlying medical conditions, cause of ECMO, mode of ECMO (venoarterial [VA]-ECMO or venovenous [VV]-ECMO), ECMO settings, and total fluid input and output on each day of ECMO therapy. Laboratory data, including white blood cell count, hemoglobin, sodium, potassium, bicarbonate, albumin, bilirubin, C-reactive protein, blood urea nitrogen, creatinine, and arterial blood gas analysis on the day of ECMO implantation, were also collected.

Acute Physiology and Chronic Health Evaluation (APACHE) II scores were calculated on the basis of medical records. The ENCOURAGE (Prediction of cardiogenic shock outcome for AMI patients salvaged by VA-ECMO) and PRESERVE (Predicting death for severe ARDS on VV-ECMO) scores were also calculated for patients in the CVD and non-CVD groups, respectively [26, 27]. The Modification of Diet in Renal Disease equation was used to determine the estimated glomerular filtration rate (eGFR) [28]. Baseline serum creatinine was defined as the average serum creatinine within 3 months prior to ICU admission. For those whose baseline creatinine levels were not obtainable, urine output was used for AKI diagnosis. Development of AKI within 72 h of ECMO implantation was evaluated using the Kidney Disease: Improving Global Outcomes (KDIGO) AKI criteria [29]. The amount of fluid administered was decided by the intensive care specialists. Crystalloid fluids were used to maintain fluid therapy for all patients. The low-chloride fluid resuscitation strategy had not been widely practiced in the institutions during the study period. Cumulative input was defined as total administration of fluid within 3 days of ECMO initiation, which was divided by patients’ body weight at the time of ECMO initiation. In addition, cumulative total output was defined as the sum of all fluid drainage from patients, and cumulative urine output as the sum of the total amount of urine during 3 days from ECMO initiation divided by body weight. CFB was defined as cumulative input minus cumulative total output. Daily fluid balance before ECMO was defined as total fluid input minus total output from ICU admission to ECMO initiation, which was divided by ICU length of stay (LOS) and patients’ body weight. If patients were admitted to the ICU after surgery, fluid balance during surgery was also included in daily fluid balance before ECMO.

### ECMO protocol

The decision to initiate ECMO therapy was made by the treating intensive care specialist or the attending cardiothoracic surgeons. Indication for ECMO was classified into two categories. Indications for ECMO in the CVD group were as follows:

1. Postcardiotomy status
2. Acute cardiogenic shock for coronary arterial disease
3. Acute myocarditis
4. Bridge to cardiac transplant for decompensated heart failure
5. Post-cardiac arrest status

Indications for ECMO in the non-CVD group were as follows:

1. Acute respiratory distress syndrome (ARDS) of any cause
2. Bridge to lung transplant
3. Miscellaneous or unknown origin

Cannulations for all ECMO supports were performed by cardiovascular surgeons with limited cut-down using the Seldinger technique [30]. Either the CAPIOX EBS (Terumo Co., Ltd., Tokyo, Japan) or the QUADROX PLS (Maquet GmbH, Rastatt, Germany) system was used in all patients. The sweep gas flow was set to maintain a partial pressure of carbon dioxide target of 35–45 mmHg under lung rest ventilation strategy. The target ECMO blood pump speed was 3.0–4.5 L/min. Systemic anticoagulation with unfractionated heparin was titrated to maintain activated clotting times (ACT) between 180 and 220 s and between 160 and 180 s for VA-ECMO and VV-ECMO, respectively. ACT was measured using STA R MAX® hemostasis analyzer (Diagnostica Stago, Inc., Parsippany, NJ, USA). Hemolysis markers, such as free plasma hemoglobin and haptoglobin, were monitored every day. Careful inspections were made by the intensive care specialist to determine the cause of hemolysis when plasma hemoglobin levels were greater than 50 mg/dl. ECMO circuit change was performed when ECMO-related technical problems were suspected as the cause of hemolysis. Red blood cell (RBC) transfusion was performed to maintain hemoglobin target between 10 and 11 g/dl. Antithrombin (AT) replacement was performed when AT activities were less than 70%. Other specific ECMO run protocols are delineated in Additional file 1: Table S1.

### Continuous renal replacement therapy protocol

In the event of the development of AKI, the decision to start continuous renal replacement therapy (CRRT) was made by the attending nephrologist. Generally, CRRT was applied to patients with AKI who had uncontrolled fluid overload, hyperkalemia, or metabolic acidosis. Continuous venovenous hemodiafiltration was applied using Prisma (Gambro Co., Ltd., Hechingen, Germany), Prismaflex (Gambro Co., Ltd.), or multiFiltrate (Fresenius Medical Care GmbH, Bad Homburg, Germany) machines. Biocompatible AN69 ST membranes were used for Prisma and Prismaflex, and polysulfone membranes were used for multiFiltrate. Bicarbonate-containing replacement fluid was administered by the predilution method. CRRT machines were routinely applied via separate lines from the ECMO circuit. However, when additional venous access was not obtainable, CRRT was performed over the same circuit with ECMO. In these cases, CRRT was applied between ECMO pump and oxygenator to avoid air entrainment.

### Clinical outcome

The patients were followed until their last visit at our centers or death. The primary endpoint was death from any cause within 90 days after ECMO initiation. Survival data were collected through electronic medical records of the hospital and outpatient clinics.

### Statistical analysis

Patients in each group were categorized into quartiles according to the CFB of each group. Continuous variables were expressed as mean ± SD. Comparisons of continuous variables were conducted with one-way analysis of variance, and linear trend was analyzed among groups. The normality of the distribution was analyzed using the Kolmogorov-Smirnov test. Variables not normally distributed were expressed as median and IQR and compared with the Jonckheere-Terpstra test. Categorical variables were expressed as percentages and compared with the chi-square test. Cumulative survival curves were derived using the Kaplan-Meier method, and statistical differences were analyzed using the log-rank test. To evaluate the association between outcomes and clinical parameters, a Cox proportional hazards model was used, and data were expressed as HR with 95% CI. This model included propensity scores. The propensity score was obtained by using a multivariate logistic regression analysis to determine whether the CFB of each patient was higher than the median amount. The covariates for adjustment were selected using a stepwise procedure when the *P* value for each variable was less than 0.2. All variables, except those that were already included in the APACHE II score, were included for propensity score calculation. Those who were lost to follow-up were treated as censored in the survival analysis. Cubic spline curve analysis was conducted as previously described by Canaud et al. [31]. The Cox model for 90-day mortality was used, and cubic spline curves had four equally distributed knots. The threshold of CFB was defined as the point where the lower limit of the 95% CI was higher than 1.0. Analyses were conducted for the CVD and non-CVD groups. Additional evaluations were also conducted after dividing the non-CVD group into a respiratory group and an others group. The respiratory group was defined as patients who underwent ECMO for lung transplant, ARDS, or other pulmonary diseases. All other patients in the non-CVD group were defined as the others group. Statistical significance was defined as *P* < 0.05. All analyses were conducted using SPSS version 23.0 software (IBM, Armonk, NY, USA) and R language (version 3.3.1; R Foundation for Statistical Computing, Vienna, Austria).

## Results

### Baseline characteristics of patients

The baseline characteristics of the patients in the CVD group are presented in Table 1. The mean age was 58.4 ± 17.7 years, and 68.2% were male. The most common cause of ECMO implantation was nonoperative CVD, and 98% of patients underwent VA-ECMO. The median CFB was 64.7 ml/kg, whereas the median daily fluid balance throughout the entire period of ECMO was 26.2 ml/kg/d. The median duration of ECMO treatment was 3 days. The age of the patients tended to increase in patients with higher CFB quartiles. Body weight tended to decrease in higher CFB quartiles, but body mass index (BMI) was comparable among the quartiles. In the non-CVD group, the mean age was 55.7 ± 15.7 years, and 65.3% of patients were male. Among these patients, 201 (63.8%) patients received ECMO treatment for respiratory disease, and VA-ECMO was applied for 23% of the patients. The median CFB was 53.5 ml/kg, and median daily fluid balance during the entire ECMO period was 15.9 ml/kg. Patients’ weight and BMI were comparable among the CFB quartiles. However, APACHE II scores tended to increase in patients with higher CFB quartiles (Table 2). In the non-CVD group, there were no significant differences in Charlson comorbidity index (*P* = 0.616), APACHE II score (*P* = 0.302), and CFB (*P* = 0.206) between VA-ECMO- and VV-ECMO-treated patients. Regarding the resuscitation volume differences during the study duration, further analyses were made by categorizing the patients into three groups according vintage: 2005–2008, 2009–2012, and 2013–2016. When the CFB quartiles were compared among the vintage groups, no statistical differences were found (Additional file 2: Figure S1).

**Table 1:** Baseline characteristics of patients with cardiovascular disease

Variables	CFB quartiles				Total	*P* for trend
	Quartile 1	Quartile 2	Quartile 3	Quartile 4		
Number of patients	101	102	102	101	406	
Age (yr)	55.0 ± 17.0	56.5 ± 18.0	58.9 ± 19.3	63.1 ± 15.5	58.4 ± 17.7	0.001
Sex (male, %)	74 (73.3)	73 (71.6)	71 (69.6)	59 (58.4)	277 (68.2)	0.025
DM (n, %)	38 (37.6)	36 (35.3)	30 (29.4)	30 (29.7)	134 (33.0)	0.156
Hypertension (n, %)	48 (47.5)	41 (40.2)	35 (34.3)	45 (44.6)	169 (41.6)	0.499
Malignancy (n, %)	6 (5.9)	13 (12.7)	9 (8.8)	7 (6.9)	35 (8.6)	0.937
Charlson comorbidity index	2.1 ± 2.1	2.2 ± 2.0	1.8 ± 1.7	1.1 ± 1.5	1.8 ± 1.9	< 0.001
Weight (kg)	67.6 ± 11.2	66.0 ± 12.9	63.9 ± 12.1	61.4 ± 10.9	64.7 ± 12.0	< 0.001
Body mass index (kg/m^2^)	24.2 ± 3.3	23.7 ± 3.8	23.4 ± 3.6	23.4 ± 3.3	23.7 ± 3.5	0.085
Body surface area (m^2^)	1.7 ± 0.2	1.7 ± 0.2	1.7 ± 0.2	1.6 ± 0.1	1.7 ± 0.2	< 0.001
CFB (ml/kg)*	− 4.8 (− 25.2–9.6)	38.2 (27.6–51.4)	109.7 (77.4–137.3)	222.9 (194.6–272.7)	64.7 (20.1–161.7)	< 0.001
Daily fluid balance (ml/kg/d)*^,†^	2.3 (− 5.0 to 8.9)	13.1 (6.6–27.7)	37.8 (23.8–67.0)	108.4 (63.4–180.6)	26.2 (6.7–68.6)	< 0.001
Cumulative input (ml/kg)*^,¶^	165.2 (124.7–216.7)	191.6 (168.3–241.8)	248.6 (213.4–280.2)	371.3 (293.3–482.3)	214.5 (157.6–267.2)	< 0.001
Cumulative total output (ml/kg)*^,¶^	182.0 (138.2–229.7)	149.7 (112.7–201.4)	138.6 (113.6–182.0)	136.8 (91.1–226.3)	153.1 (115.6–201.4)	< 0.001
Cumulative urine output (ml/kg)*^,¶^	136.0 (68.5–182.5)	100.5 (37.1–143.9)	59.8 (21.7–119.8)	27.1 (10.0–66.6)	95.8 (31.1–150.6)	< 0.001
ICU LOS before ECMO (days)*	1 (0–1)	1 (0–1)	0 (0–1)	0 (0–1)	1 (0–1)	< 0.001
Daily fluid balance before ECMO (ml/kg/d)*^,‡^	1.4 (− 22.4–19.9)	11.6 (− 15.7–24.4)	11.9 (− 0.6–37.9)	− 0.7 (− 6.2–7.4)	3.1 (− 8.3–21.6)	0.834
ECMO cause (n, %)						
Heart transplant	5 (5.0)	9 (8.8)	2 (2.0)	0 (0.0)	16 (3.9)	0.357
Other cardiotomy	22 (21.8)	23 (22.5)	20 (19.6)	21 (20.8)	86 (21.2)	
Nonoperative CVD	74 (73.3)	70 (68.6)	80 (78.4)	80 (79.2)	304 (74.9)	
ECMO VA mode (n, %)	100 (99.0)	98 (96.1)	99 (97.1)	101 (100.0)	398 (98.0)	0.530
ECMO settings						
Blood flow rate (L/min)	2.8 ± 0.8	3.0 ± 0.8	3.0 ± 1.0	3.1 ± 1.0	2.9 ± 0.9	0.006
Blood flow rate (L/min/m^2^)	1.6 ± 0.5	1.8 ± 0.4	1.8 ± 0.6	1.9 ± 0.6	1.8 ± 0.5	< 0.001
FsO_2_ (%)	89.4 ± 13.8	87.9 ± 15.1	85.3 ± 15.8	81.3 ± 17.1	85.9 ± 15.7	< 0.001
ECMO DO_2_I (ml/min/m^2^)	272.4 ± 86.7	304.4 ± 101.6	311.9 ± 134.6	344.8 ± 134.9	309.3 ± 119.2	< 0.001
ECMO duration (days)*	4 (2–6)	3 (2–7)	4 (2–7)	3 (2–6)	3 (2–6)	0.231
Mechanical ventilation (n, %)	90 (89.2)	100 (98.5)	97 (95.3)	100 (98.9)	387 (95.3)	0.015
Days of ventilator before ECMO (days)*	1 (0–1)	1 (0–1)	1 (0–1)	0 (0–1)	1 (0–1)	0.155
WBC (× 10^3^/μl)	12.8 ± 6.0	13.8 ± 6.9	14.6 ± 8.5	13.6 ± 6.8	13.7 ± 7.1	0.293
Hemoglobin (g/dl)	11.1 ± 2.4	11.4 ± 2.4	11.0 ± 2.3	10.3 ± 2.4	11.0 ± 2.4	0.008
SaO_2_ (%)*	98.2 (93.3–99.3)	97.7 (95.4–98.9)	99.3 (97.3–99.8)	99.6 (96.8–99.9)	98.8 (96.2–99.8)	0.001
BUN (mg/dl)	27.3 ± 16.0	27.1 ± 15.2	26.5 ± 11.0	23.6 ± 12.9	26.1 ± 14.0	0.054
Creatinine (mg/dl)	1.5 ± 0.9	1.5 ± 0.8	1.5 ± 0.5	1.4 ± 0.5	1.5 ± 0.7	0.105
eGFR (ml/min/1.73 m^2^)	60.7 ± 29.5	58.2 ± 26.8	54.6 ± 22.9	58.2 ± 25.8	57.9 ± 26.4	0.348
Albumin (g/dl)	3.0 ± 0.5	2.9 ± 0.6	2.7 ± 0.7	2.6 ± 0.8	2.8 ± 0.7	< 0.001
Bilirubin (mg/dl)	1.8 ± 1.7	2.4 ± 2.6	1.8 ± 1.9	1.2 ± 1.2	1.8 ± 1.9	0.007
CRP (mg/dl)*	32.7 (12.8–82.6)	25.6 (6.4–62.6)	17.9 (4.1–61.9)	9.7 (3.0–47.0)	22.7 (3.6–62.3)	0.001
APACHE II score	23.1 ± 9.7	23.9 ± 8.3	23.7 ± 10.4	24.7 ± 9.7	23.8 ± 9.6	0.274

*Abbreviations: APACHE II* Acute Physiology and Chronic Health Evaluation II, *ARDS* Acute respiratory distress syndrome, *BUN* Blood urea nitrogen, *CFB* Cumulative fluid balance for 3 days from extracorporeal membrane oxygenation initiation, *CRP* C-reactive protein, *CVD* Cardiovascular disease, *DM* Diabetes mellitus, *ECMO* Extracorporeal membrane oxygenation, *eGFR* Estimated glomerular filtration rate, *FsO*_*2*_ Fraction of sweep gas inlet oxygen supplied to extracorporeal membrane oxygenation, *SaO*_*2*_ Oxygen saturation, *VA* Venoarterial access, *WBC* White blood cell

*Data are expressed as median and IQR and compared by Jonckheere-Terpstra test

^†^Fluid balance during entire ECMO treatment

^¶^During 3 days from ECMO commencement

^‡^Daily fluid balance during ICU admission before ECMO commencement

**Table 2:** Baseline characteristics of patients with non-cardiovascular disease

Variables	CFB quartiles				Total	*P* for trend
	Quartile 1	Quartile 2	Quartile 3	Quartile 4		
Number of patients	79	79	80	79	317	
Age (yr)	53.2 ± 16.2	56.1 ± 14.9	57.2 ± 15.0	56.3 ± 16.8	55.7 ± 15.7	0.192
Sex (male, %)	55 (69.6)	53 (67.1)	52 (65.0)	47 (59.5)	207 (65.3)	0.176
DM (n, %)	19 (24.1)	17 (21.5)	15 (18.8)	16 (20.3)	67 (21.1)	0.490
Hypertension (n, %)	21 (26.6)	13 (16.5)	14 (17.5)	21 (26.6)	69 (21.8)	0.962
Malignancy (n, %)	25 (31.6)	22 (27.8)	22 (27.5)	24 (30.4)	93 (29.3)	0.856
Charlson comorbidity index	2.1 ± 2.2	1.9 ± 2.0	1.6 ± 1.7	1.6 ± .2.0	1.8 ± 2.0	0.076
Weight (kg)	63.8 ± 15.8	63.1 ± 12.8	64.1 ± 16.1	61.0 ± 12.2	63.0 ± 14.3	0.300
Body mass index (kg/m^2^)	22.9 ± 4.4	23.4 ± 4.5	23.6 ± 5.1	22.7 ± 3.9	23.2 ± 4.5	0.900
Body surface area (m^2^)	1.7 ± 0.2	1.7 ± 0.2	1.6 ± 0.2	1.6 ± 0.2	1.7 ± 0.2	0.151
CFB (ml/kg)*	− 3.9 (− 19.8–6.8)	34.5 (25.6–42.7)	72.5 (60.2–87.5)	194.7 (147.8–265.5)	53.5 (16.9–106.7)	< 0.001
Daily fluid balance (ml/kg/d)*^,†^	4.3 (− 2.7–12.9)	12.8 (7.1–19.7)	21.1 (11.2–35.9)	54.0 (29.5–101.8)	15.9 (7.1–36.4)	< 0.001
Cumulative input (ml/kg)*^,¶^	195.5 (165.3–264.7)	206.4 (166.2–263.6)	246.2 (192.4–292.3)	338.6 (271.8–422.5)	231.8 (180.8–298.8)	< 0.001
Cumulative total output (ml/kg) *^,¶^	206.6 (163.9–274.9)	166.2 (132.2–221.7)	173.7 (126.7–217.0)	127.7 (92.0–212.9)	183.2 (130.4–233.9)	< 0.001
Cumulative urine output (ml/kg) *^,¶^	107.2 (8.1–180.9)	98.3 (13.2–163.5)	75.7 (15.6–127.8)	42.7 (17.3–109.8)	83.9 (14.0–159.6)	0.055
ICU LOS before ECMO (days)*	2.5 (0–6)	2 (1–4)	1 (0–5)	1 (0–5)	2 (0–5)	0.636
Daily fluid balance before ECMO (ml/kg/d)*^,‡^	6.8 (− 4.8–18.2)	17.0 (7.6–35.6)	12.7 (4.0–45.1)	22.0 (4.0–49.8)	12.2 (3.9–32.5)	< 0.001
ECMO cause (n, %)						
Lung transplant	17 (21.5)	14 (17.9)	12 (15.2)	5 (6.3)	48 (15.2)	0.081
ARDS	17 (21.5)	19 (24.4)	14 (17.7)	25 (31.6)	75 (23.8)	
Other pulmonary	13 (16.5)	21 (26.9)	17 (21.5)	27 (34.2)	78 (24.8)	
Others	32 (40.5)	25 (31.6)	37 (46.3)	22 (27.8)	116 (36.6)	
ECMO VA mode (n, %)	18 (22.7)	12 (15.2)	22 (27.5)	21 (26.6)	73 (23.0)	0.321
ECMO settings						
Blood flow rate (L/min)	3.1 ± 0.8	3.0 ± 0.7	3.1 ± 0.8	3.1 ± 0.8	3.1 ± 0.8	0.661
Blood flow rate (L/min/m^2^)	1.8 ± 0.5	1.8 ± 0.4	1.8 ± 0.5	1.8 ± 0.5	1.8 ± 0.5	0.965
FsO_2_ (%)	91.1 ± 13.7	92.0 ± 13.7	90.7 ± 13.6	85.0 ± 16.6	89.7 ± 14.6	0.008
ECMO DO_2_I (ml/min/m^2^)	297.3 ± 87.9	302.6 ± 93.8	281.2 ± 89.5	306.0 ± 115.2	297.0 ± 97.6	0.889
ECMO duration (days)*	8 (4–16)	8 (3–14)	8 (3–12)	6 (3–12)	8 (3–13)	0.119
Mechanical ventilation (n, %)	77 (97.5)	77 (97.5)	78 (97.5)	78 (98.7)	310 (97.8)	0.785
Days of ventilator before ECMO (days)*	3 (1–4)	2 (2–3)	2 (0–3)	3 (1–5)	3 (1–3)	0.377
WBC (×10^3^/μl)	11.6 ± 6.6	12.8 ± 7.2	12.1 ± 7.9	14.0 ± 8.7	12.6 ± 7.6	0.108
Hemoglobin (g/dl)	10.8 ± 2.0	11.1 ± 2.5	10.4 ± 1.9	10.3 ± 2.5	10.7 ± 2.2	0.042
SaO_2_ (%)*	94.0 (88.2–98.8)	94.4 (90.1–98.0)	93.2 (80.7–98.5)	95.3 (89.3–99.0)	94.4 (88.0–98.7)	0.707
BUN (mg/dl)	31.2 ± 20.6	26.3 ± 16.9	27.2 ± 14.8	29.5 ± 25.4	28.5 ± 19.9	0.684
Creatinine (mg/dl)	1.4 ± 1.0	1.2 ± 0.8	1.3 ± 0.9	1.1 ± 0.9	1.3 ± 0.9	0.305
eGFR (ml/min/1.73 m^2^)	89.9 ± 62.7	94.3 ± 61.0	77.7 ± 54.8	90.6 ± 51.4	88.1 ± 57.7	0.622
Albumin (g/dl)	2.7 ± 0.6	2.8 ± 0.6	2.6 ± 0.7	2.5 ± 0.7	2.7 ± 0.6	0.026
Bilirubin (mg/dl)	2.9 ± 3.7	3.4 ± 5.4	3.0 ± 3.8	1.9 ± 2.0	2.8 ± 3.9	0.108
CRP (mg/dl) *	69.7 (28.1–155.2)	74.7 (32.2–141.6)	85.0 (17.9–199.1)	99.2 (22.0–170.8)	81.2 (23.7–176.8)	0.735
APACHE II score	18.9 ± 7.3	20.0 ± 7.3	19.6 ± 7.7	24.1 ± 8.7	20.7 ± 8.0	< 0.001

*Abbreviations: APACHE II* Acute Physiology and Chronic Health Evaluation II, *ARDS* Acute respiratory distress syndrome, *BUN* Blood urea nitrogen, *CFB* Cumulative fluid balance for 3 days from extracorporeal membrane oxygenation initiation, *CRP* C-reactive protein, *CVD* Cardiovascular disease, *DM* Diabetes mellitus, *ECMO* Extracorporeal membrane oxygenation, *eGFR* Estimated glomerular filtration rate, *FsO*_*2*_ Fraction of sweep gas inlet oxygen supplied to extracorporeal membrane oxygenation, *SaO*_*2*_ Oxygen saturation, *VA* Venoarterial access, *WBC* White blood cell

*Data are expressed as median and IQR and compared by Jonckheere-Terpstra test

^†^Fluid balance during entire ECMO treatment

^¶^During 3 days from ECMO commencement

^‡^Daily fluid balance during ICU admission before ECMO commencement

### Outcome of patients

In the CVD group, AKI occurred in 306 (75.4%) patients and CRRT was commenced in 127 (31.3%) patients during the initial 3 days of ECMO initiation. The occurrence rate of AKI tended to increase in quartiles with higher CFB values. In addition, in the CVD group, median ICU LOS was 8 days. A total of 207 deaths (51.0%) occurred within 90 days of ECMO initiation, and the mortality rate tended to increase in higher CFB quartiles (*P* < 0.001). In the non-CVD group, 184 (70.0%) patients underwent AKI, and CRRT was applied in 92 (29.0%) patients during first 3 days of ECMO commencement. Both AKI occurrence (*P* = 0.011) and CRRT application rates (*P* = 0.011) tended to increase in patients with higher CFB. Patients treated with VA-ECMO had a significantly higher incidence of AKI than those treated with VV-ECMO (*P* = 0.014). The median ICU LOS was 16 days. During the 90 days of ECMO initiation, a total of 209 (65.9%) deaths occurred (Table 3).

**Table 3:** Outcome of patients according to cumulative fluid balance quartiles

Variables	CFB quartiles				Total	*P* for trend
	Quartile 1	Quartile 2	Quartile 3	Quartile 4		
Cardiovascular disease						
Incident AKI (n, %)	60 (59.3)	76 (74.4)	86 (84.3)	84 (83.1)	306 (75.4)	< 0.001
CRRT treatment (n, %)*	26 (25.7)	24 (23.5)	48 (47.1)	29 (28.7)	127 (31.3)	0.114
Days of CRRT before ECMO (days)^†^	0 (0–1)	0 (0–1)	1 (0–1)	0 (0–2)	1 (0–1)	0.082
ICU LOS (days)^†,‡^	10 (5–16)	10 (6–18)	7 (3–14)	7 (4–14)	8 (4–15)	0.014
90-day mortality (n, %)	35 (34.7)	43 (42.2)	71 (69.6)	58 (57.4)	207 (51.0)	< 0.001
Non-cardiovascular disease						
Incident AKI (n, %)	47 (68.1)	34 (53.1)	54 (76.1)	49 (83.1)	184 (70.0)	0.011
CRRT treatment (n, %)*	17 (21.5)	16 (20.3)	32 (40.0)	27 (34.2)	92 (29.0)	0.011
Days of CRRT before ECMO (days)^†^	1 (0–2)	1 (1–2)	2 (1–2)	1 (1–2)	3 (1–3)	0.483
ICU LOS (days)^†,‡^	18 (10–33)	17 (11–28)	13 (8–31)	17 (6–40)	16 (9–32)	0.239
90-day mortality (n, %)	49 (62.0)	43 (54.4)	63 (78.8)	54 (68.4)	209 (65.9)	0.069

*Abbreviations: AKI* Acute kidney injury, *CFB* Cumulative fluid balance, *CRRT* Continuous renal replacement therapy, *ECMO* Extracorporeal membrane oxygenation, *ICU* Intensive care unit, *LOS* Length of stay

*Continuous renal replacement therapy imitation during the first 3 days of ECMO commencement

^†^Data are expressed as median and IQR and compared by Jonckheere-Terpstra test

^‡^LOS was presented in patients who survived at 30th day from ECMO initiation

### Relationship between CFB quartile groups and mortality

**Fig. 2:**
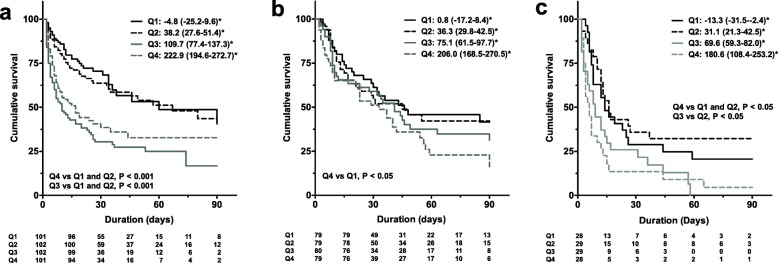
Kaplan-Meier (KM) plots for 90-day mortality in each cumulative fluid balance quartile. **a** KM plots representing patients with cardiovascular disease. **b** KM plots representing patients with respiratory disease. **c** KM plots representing patients with other disease. *Median and IQR of cumulative fluid balance (ml/kg) for each quartile

The Kaplan-Meier curves revealed that the cumulative survival rates of CFB quartiles 3 (*P* < 0.001) and 4 (*P* < 0.001) were significantly lower than those of CFB quartiles 1 and 2 in the CVD group (Fig. 2a). The non-CVD group was further divided into those initiating ECMO for respiratory failure (respiratory group) and other causes (others group). The characteristics of the respiratory group are presented in Additional file 1: Table S2. In patients in the respiratory group, the cumulative survival rate of CFB quartile 4 was significantly lower than quartile 1 (*P* = 0.047) (Fig. 2b). In addition, in the others group, CFB quartile 4 showed a significantly lower cumulative survival rate than quartile 1 (*P* = 0.004) and quartile 2 (*P* = 0.001) (Fig. 2c). When patients in the non-CVD group were divided into those treated with VA-ECMO or VV-ECMO, those treated with VA-ECMO had significantly lower cumulative survival rates than those treated with VV-ECMO (*P* = 0.002).

In addition, when the association between CFB and mortality was further evaluated using multivariate Cox proportional hazards regression analyses, in the CVD group, the risk of mortality significantly increased in patients in CFB quartiles 3 (HR, 2.58; 95% CI, 1.62–4.11; *P* < 0.001) and 4 (HR, 2.11; 95% CI, 1.26–3.54; P = 0.004) compared with that of patients in CFB quartile 1. However, the mortality risk did not show a significant increase among patients in CFB quartile 2. Similarly, in the non-CVD group, the mortality risk of CFB quartiles 3 (HR, 1.66; 95% CI, 1.06–2.59; *P* = 0.026) and 4 (HR, 1.69, 95% CI, 1.05–2.72; *P* = 0.03) was significantly higher than quartile 1, whereas mortality risk did not significantly differ between CFB quartiles 1 and 2 (Table 4). These findings imply that a CFB below the level of CFB quartile 3 in both the CVD and non-CVD groups may not increase the risk of mortality. When CFB was considered as a continuous variable, log-transformed CFB was independently associated with 90-day mortality in both the CVD group (HR, 1.76; 95% CI, 1.37–2.27; *P* < 0.001) and the non-CVD group (HR, 1.46; 95% CI, 1.17–1.83; *P* < 0.001) (Table 5).

**Table 4:** Cox regression analyses for 90-day mortality

CFB quartiles	Cardiovascular disease				Non-cardiovascular disease			
	Unadjusted		Adjusted*		Unadjusted		Adjusted*	
	HR (95% CI)	*P* value	HR (95% CI)	*P* value	HR (95% CI)	*P* value	HR (95% CI)	*P* value
Quartile 1	1.00 (reference)		1.00 (reference)		1.00 (reference)		1.00 (reference)	
Quartile 2	1.17 (0.75–1.83)	0.496	1.27 (0.77–2.10)	0.342	0.84 (0.56–1.27)	0.407	0.79 (0.48–1.30)	0.345
Quartile 3	2.82 (1.88–4.23)	< 0.001	2.58 (1.62–4.11)	< 0.001	1.61 (1.11–2.34)	0.012	1.66 (1.06–2.59)	0.026
Quartile 4	2.24 (1.47–3.41)	< 0.001	2.11 (1.26–3.54)	0.004	1.37 (0.93–2.02)	0.108	1.69 (1.05–2.72)	0.030

*CFB* Cumulative fluid balance

*Adjusted for age, sex, Charlson comorbidity index, Acute Physiology and Chronic Health Evaluation II score, and propensity score

Propensity score was obtained by logistic regression analysis with covariables body mass index, extracorporeal membrane oxygenation (ECMO) pump time, ECMO blood flow rate, albumin, total carbon dioxide, acute kidney injury stage

The 27 (3.7%) patients who were lost to follow-up were treated as censored

**Table 5:** Cox regression analyses for 90-day mortality with cumulative fluid balance, cumulative input, and output

Models	Variables	Cardiovascular disease		Non-cardiovascular disease	
		HR (95% CI)	*P* value	HR (95% CI)	*P* value
Model 1	CFB*	1.76 (1.37–2.27)	< 0.001	1.46 (1.17–1.83)	< 0.001
Model 2	CFB*	1.67 (1.19–2.34)	0.003	1.55 (1.16–2.09)	0.003
Model 3	Cumulative input*^,†^	3.35 (1.64–6.83)	0.001	5.53 (2.60–11.75)	< 0.001
	Cumulative total output*^,†^	0.56 (0.47–0.76)	< 0.001	0.25 (0.14–0.45)	< 0.001
Model 4	Cumulative input*^,†^	2.66 (1.30–5.44)	0.007	1.92 (1.08–3.42)	0.027
	Cumulative urine output*^,†^	0.78 (0.67–0.91)	0.001	0.84 (0.75–0.93)	0.001

*CFB* Cumulative fluid balance

Model 1: Additionally adjusted for age, sex, Charlson comorbidity index, Acute Physiology and Chronic Health Evaluation II score, and propensity score

Model 2: Model 1 + daily fluid balance before ECMO*^,¶^

Model 3: Model 2 + cumulative input + cumulative total output without CFB

Model 4: Model 2 + cumulative input + cumulative urine output without CFB

*Data were log-transformed

^†^During 3 days from extracorporeal membrane oxygenation (ECMO) commencement

^¶^Daily fluid balance during intensive care unit admission before ECMO commencement

Propensity score was obtained by logistic regression analysis with covariables body mass index, ECMO pump time, ECMO blood flow rate, albumin, total carbon dioxide, acute kidney injury stage

The 27 (3.7%) patients who were lost to follow-up were treated as censored

No significant interactions between APACHE II scores and CFBs were found in both the CVD and non-CVD groups (*P* = 0.521 for CVD group, *P* = 0.166 for non-CVD group). When further adjustments were made for daily fluid balance before ECMO commencement, the relationship between CFB and risk of mortality was preserved in both groups. In addition, cumulative input was independently associated with 90-day mortality even after adjustments were made for cumulative total output and urine output (Table 5).

When multivariate Cox models were analyzed with adjustment for ENCOURAGE score instead of APACHE II score in the CVD group, the relationship between CFB and 90-day mortality remained significant. This relationship was also maintained when adjustments were made for PRESERVE score instead of APACHE II score in patients who had undergone VV-ECMO in the non-CVD group (Additional file 1: Table S3).

### Significant CFB threshold level in ECMO patients

**Fig. 3:**
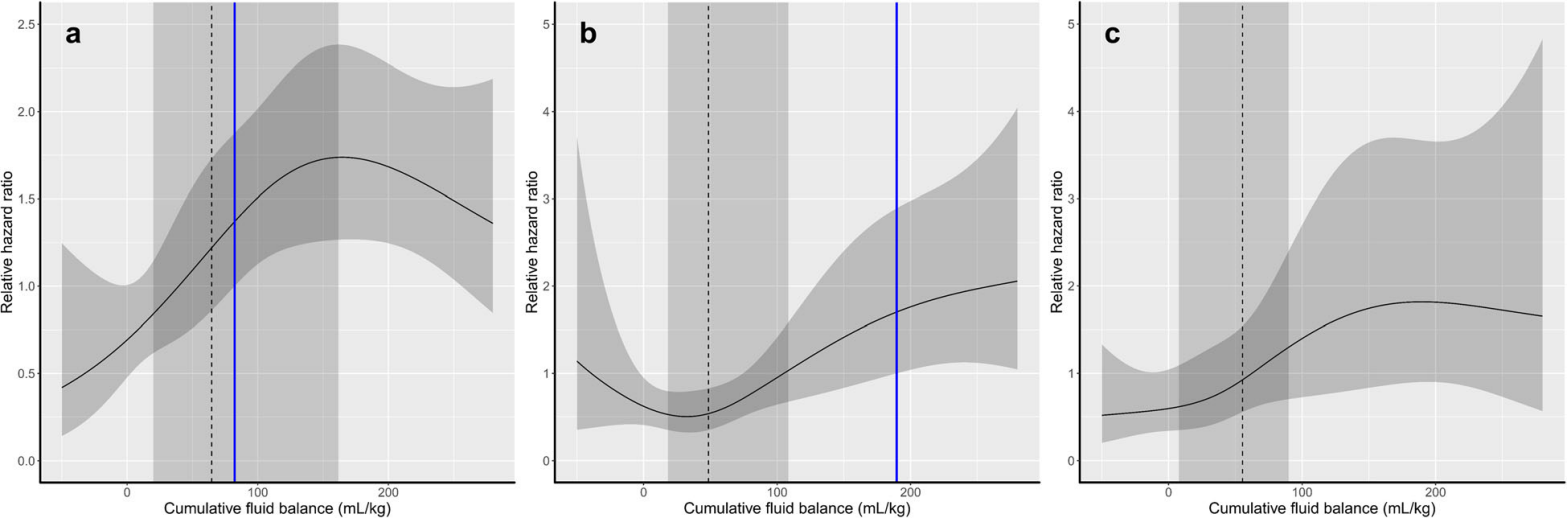
Cubic spline curves for the association between cumulative fluid balance and 90-day mortality. **a** Cubic spline curve representing patients with cardiovascular disease. **b** Cubic spline curve representing patients with respiratory disease. **c** Cubic spline curve representing patients with other disease. *Dashed line* = median cumulative fluid balance, *dark gray area* = IQR, *blue line* = permissive threshold of cumulative fluid balance

To further investigate the CFB threshold level that considerably increases the risk of mortality in ECMO patients, the relationship between CFB and mortality risk was evaluated by cubic spline analyses. In the CVD group, the relative HR of mortality started to increase significantly when CFB values were greater than 82.3 ml/kg (Fig. 3a). The relative HR increased significantly above CFB levels of 189.6 ml/kg in the respiratory group (Fig. 3b). In the others group, relative HR tended to increase with higher CFB amounts without statistical significance (Fig. 3c).

## Discussion

Large amounts of fluid resuscitation are inevitable in patients initiating ECMO. However, excessive fluid overload can negatively impact outcomes in these patients. In this study, we have shown that CFB was a significant factor affecting mortality risk in patients treated with ECMO. This increase in mortality risk was significant only when the CFB was greater than 82.3 ml/kg in patients who were treated with ECMO for cardiovascular reasons. For those who were treated with ECMO for respiratory causes, mortality risk increase was substantial when CFB was above 189.6 ml/kg.

In patients undergoing ECMO treatment, large-volume fluid administration is frequently required. This is due mainly to disease severity. However, maintaining adequate blood flow for ECMO treatment also plays a part. Patients typically go through a systemic inflammatory response within the first few days of ECMO treatment [6]. This response induces pathologic vasodilation and fluid loss to the interstitial compartment, resulting in reduced vascular volume. In addition, major conditions associated with patients undergoing ECMO, such as shock and low cardiac output as well as increased capillary leakage related to sepsis-like syndrome, are factors that contribute [8, 32, 33]. This insufficient intravascular volume can lead to extracorporeal flow failure, which results in more frequent ECMO circuit changes and decreased total ECMO delivery time [6, 34]. In a retrospective study of pediatric patients who received ECMO treatment for severe pneumonia, frequent circuit change was a significant risk factor for death [35]. In addition, in a recent investigation of 172 adult patients receiving ECMO, ECMO circuit change was more frequent in patients undergoing concomitant CRRT and ECMO treatment, which resulted in an increased mortality rate in these patients [19]. Therefore, the administration of sufficient amounts of intravenous fluid to maintain a satisfactory extracorporeal blood flow is a critical component in the management of patients initiating ECMO. Accordingly, in this study, 85.9% of the patients maintained a positive CFB. In addition, the overall average CFB was greater than that of previously reported ICU patients not undergoing ECMO treatment [15, 21].

Excessive CFB during the initial phase of ECMO was found to be independently associated with increased mortality risk. This association between positive fluid balance and survival in ECMO-treated patients has been proposed previously. In a study of 172 patients receiving VA-ECMO or VV-ECMO treatment, positive fluid balance at the third day of ECMO treatment was an independent predictor of mortality [19]. In addition, in pediatric patients requiring concomitant ECMO and CRRT treatment, the degree of fluid overload at the time of ECMO initiation was significantly associated with mortality [36]. In this study, the CFB showed a significant independent relationship with 90-day mortality. However, interestingly, the risk of mortality did not show a significant increase at CFB levels less than 82.3 ml/kg and 189.6 ml/kg for the CVD and respiratory groups, respectively. This finding suggests that patients undergoing ECMO might be able to tolerate fluid replacement below these thresholds. The risk of mortality was elevated in proportion to increases in the CFB above these thresholds, thereby confirming the relationship between CFB and mortality risk suggested in previous investigations [19, 20]. However, the relationships between mortality and CFB were not linear. As mentioned above, decreased intravascular volume associated with inadequate fluid resuscitation could cause frequent ECMO circuit change, consequently affecting outcome [6, 34]. This may be one of the reasons why mortality risk did not show a linear relationship at lower CFB levels. In the CVD group, HR of mortality did not seem to increase linearly when CFB was greater than 150 ml/kg. However, the range of the CIs was too wide to infer a significant relationship. This could be due to the fact that the number of patients with extremely high CFB was relatively small. The threshold CFB amounts proposed in this study require further validation. However, in light of the fact that a positive fluid balance during the initial phase of ECMO treatment is generally unavoidable, the CFB thresholds suggested herein, as determined by mortality risk, could serve as a basis for the definition of the clinically significant fluid overload threshold in ECMO-treated patients.

The increased CFB in this study could have been a result of disease severity, considering that those with severe systemic vascular leakage and decreased cardiac function would need larger amounts of fluid resuscitation. The APACHE II scores were not significantly different between CFB quartiles in the CVD group. However, an APACHE II score increase was noticed in the highest quartile of the non-CVD group. Fluid overload may be a result of severe patient conditions, but it could also affect outcome by aggravating vascular wall stretching and worsening vascular permeability [37]. Because fluid balance and patient severity are interconnected during the treatment course of critically ill patients, it is not easy to delineate a causal relationship [37]. Accordingly, in the current study, owing to the retrospective nature of the investigation, defining the exact causality between severity and fluid balance was not possible. However, even after adjustments were made for disease severity, a significant increased risk of mortality was noted in the higher CFB quartiles, suggesting that fluid balance could have played an independent role in affecting outcome. Additionally, the fact that no significant interactions were found between disease severity and fluid balance further suggests the possibility that the results presented in this study were an effect of fluid balance rather than disease severity. Nevertheless, further investigations controlling the resuscitation volume amount would be needed to further confirm the findings of this study.

Both decreased output and increased input played a role in increasing CFB in ECMO-treated patients in this study. Lower cumulative total output and cumulative urine output were both independently associated with increased risk of mortality. Meanwhile, higher cumulative input also had a significant relationship with mortality. Notably, this significant relationship with cumulative input was robust even after adjustments were made for cumulative total output or cumulative urine output. Therefore, although decreased output contributes to the positive CFB, increased fluid input still plays a significant role in affecting outcome. Moreover, cumulative input clearly increases the risk of mortality, regardless of output amount, suggesting that the positive input may affect outcome independently of disease severity.

The CFB threshold levels found in this study were higher in the non-CVD group than in the CVD group. The fact that the patients in the non-CVD group were younger and had lower APACHE II scores than those in the CVD group opens the possibility that the non-CVD group could have been healthier and thereby able to tolerate larger amounts of CFB than the CVD group. Hemodynamic status in VV-ECMO-receiving patients is determined by a complex interaction of factors, such as intrathoracic pressure, native cardiac function, pulmonary vascular resistance, systemic vascular tone, and recirculation on VV-ECMO, in addition to intravascular volume. However, the effects of intrathoracic pressure, native cardiac function, and pulmonary vascular resistance on maintaining hemodynamic status are not substantial in patients undergoing VA-ECMO, making these patients relatively more vulnerable to volume than VV-ECMO patients. Furthermore, in VA-ECMO patients, unloading the heart with strict volume control may result in effective preload reduction. However, in VV-ECMO patients, an adequate amount of preload would be needed to maintain cardiac output. This could be one of the reasons that the permissive CFB amount was lower in the CVD group, in which most of the patients underwent VA-ECMO, than in the non-CVD group. Considering that most of the patients in the CVD group underwent VA-ECMO, ECMO modality could also have played a role in determining CFB threshold levels.

There are several limitations of this study. First, although adjustments were made for various confounding factors, including those that reflected the disease severity of the patients and the propensity scores, the limitations owing to the retrospective design should still be recognized. Second, data regarding medications such as diuretics, and hemodynamic parameters including pulmonary arterial pressure, cardiac chamber size, and cardiac output were not obtainable. Third, parameters to ensure sufficient ECMO treatment, such as oxygen consumption, RBC transfusion, amount of recirculation, incidence of catheter change, or ECMO circuit change could not be retrieved from the electronic medical records. Fourth, although protocol-based treatments were applied for all patients within each center, protocol details and bedside care would have varied somewhat from center to center and also over time. Finally, because the indications and modes of ECMO treatment varied, the results of this study may not be generalizable to all patients undergoing ECMO. Although the results were controlled for different indications and ECMO modalities, validation in specific subgroups is needed.

## Conclusions

Excessive CFB during the early phase of ECMO treatment increased the risk of mortality. However, this risk did not increase significantly until the CFB reached specific threshold ranges, which differed according to the disease origin responsible for ECMO commencement. These results suggest that fluid therapy in ECMO-treated patients should be adjusted so as not to exceed this clinically significant fluid overload level. Further interventional studies are needed to confirm these findings.

## Additional files

Additional file 1: **Table S1.** ECMO protocols for patients. **Table S2.** Baseline characteristics of patients with respiratory disease in non-CVD group. **Table S3.** Cox regression analyses for 90-day mortality with ENCOURAGE and PRESERVE scores. (DOCX 31 kb)

Additional file 2: **Figure S1.** Cumulative fluid balance according to ECMO treatment vintage. (PDF 31 kb)

## Abbreviations

ACT: Activated clotting time
AKI: Acute kidney injury
APACHE: Acute Physiology and Chronic Health Evaluation
ARDS: Acute respiratory distress syndrome
AT: Antithrombin
BMI: Body mass index
CFB: Cumulative fluid balance
CRRT: Continuous renal replacement therapy
CVD: Cardiovascular disease
ECMO: Extracorporeal membrane oxygenation
eGFR: Estimated glomerular filtration rate
ENCOURAGE: Prediction of cardiogenic shock outcome for AMI patients salvaged
ESRD: End-stage renal disease
ICU: Intensive care unit
LOS: Length of stay
PRESERVE: Predicting death for Severe ARDS on VV-ECMO
RBC: Red blood cell
VA: Venoarterial
VV: Venovenous
