# Supplementary material for: Permissive fluid volume in adult patients undergoing extracorporeal membrane oxygenation treatment
Source: Crit Care. 2018 Oct 27;22:270. doi: 10.1186/s13054-018-2211-x (PMC6203979; doi:10.1186/s13054-018-2211-x)
Supplement: Supplementary file 1 — Table S1. ECMO protocols for patients. Table S2. Baseline characteristics of patients with respiratory disease in non-CVD group. Table S3. Cox regression analyses for 90-day mortality with ENCOURAGE and PRESERVE scores. (DOCX 31 kb) [file 13054_2018_2211_MOESM1_ESM.docx]

**Table S1. ECMO protocols for patients.**

|  | **VA-ECMO** | **VV-ECMO** |
| --- | --- | --- |
| Cannulation* |  |  |
| Drainage cannula | Femoral vein (20F/53cm) | Femoral vein (21F/53cm) |
| Returning cannula | Femoral artery (16F/18cm) | Internal jugular vein (17F/53cm) |
| Tip position |  |  |
| Drainage cannula | Mid-right atrium | Junction of IVC and right atrium |
| Returning cannula | Common iliac artery | Junction of SVC and right atrium |
| Priming fluid | Hartmann’s solution | |
| ECMO blood flow | 3.0-4.5 L/min | |
| Sweep gas flow | PaCO_2_ target 35-45 mmHg | |
| Systemic anticoagulation | Unfractionated heparin | |
| ACT target | 180-220 seconds | 160-180 seconds |
| Hemolysis monitoring | Free plasma hemoglobin, haptoglobin (every day) | |
| Transfusion target | Hemoglobin level 10-11 g/dL | |
| Antithrombin III |  | |
| Monitoring | Every other day | |
| Replacement | Antithrombin III activities < 70% | |
| Ventilator settings |  | |
| Tidal volume | 3-5 mL/kg | |
| Respiratory rate | 12-16/min | |
| Plateau inspiratory pressure | < 25 cmH_2_O | |
| PEEP | - 1. mH_2_O | |

*Single stage cannulae with side pores supplied by DLP Medtronic (Minneapolis, MN, USA) were used. Cannula information was obtainable from 2013 for Seoul National University Hospital and Seoul National University Bundang Hospital, and from 2010 for Yonsei University Health System.

Abbreviations: ECMO, extracorporeal membrane oxygenation; VA, veno-arterial; VV, veno-venous; IVC, inferior vena cava; SVC, superior vena cava; ACT, activated clotting time; PEEP, positive end-expiratory pressure.

**Table S2.** Baseline characteristics of patients with respiratory disease in non-CVD group

| **Variables** | **CFB quartiles** | | | | **Total** | **P for trend** |
| --- | --- | --- | --- | --- | --- | --- |
|  | **Quartile 1** | **Quartile 2** | **Quartile 3** | **Quartile 4** |  |  |
| Number of patients | 50 | 50 | 51 | 50 | 201 |  |
| Age (yr) | 52.0±16.5 | 55.9±14.1 | 55.4±15.5 | 58.3±14.5 | 55.4±15.2 | 0.06 |
| Sex (Male, %) | 32 (64.0) | 33 (66.0) | 32 (62.7) | 31 (62.0) | 128 (63.7) | 0.761 |
| DM (n, %) | 12 (24.0) | 12 (24.0) | 10 (19.6) | 10 (20.0) | 44 (21.9) | 0.531 |
| Hypertension (n, %) | 14 (28.0) | 10 (20.0) | 7 (13.7) | 13 (26.0) | 44 (21.9) | 0.635 |
| Malignancy (n, %) | 11 (22.0) | 9 (18.0) | 10 (19.6) | 15 (30.0) | 45 (22.4) | 0.334 |
| Charlson comorbidity index | 2.3±2.4 | 1.5±1.6 | 1.7±1.7 | 1.6±2.2 | 1.8±2.0 | 0.131 |
| Weight (kg) | 62.7±16.1 | 61.2±9.9 | 58.3±10.4 | 60.4±11.3 | 60.7±12.2 | 0.207 |
| Body mass index (kg/m^2^) | 22.5±4.3 | 22.3±3.4 | 22.0±3.6 | 22.3±3.6 | 22.3±3.7 | 0.928 |
| CFB (mL/kg)*^*^*  Daily fluid balance (mL/kg/d)^*,†^ | 0.8  (-17.2-8.4)  3.9  (-4.5-9.6) | 36.3  (29.8-42.5)  13.8  (7.9-19.4) | 75.1  (61.5-97.7)  20.6  (10.0-34.0) | 206.0  (168.5-270.5)  40.5  (23.4-71.4) | 48.6  (18.2-108.4)  14.7  (6.6-32.4) | <0.001  <0.001 |
| ECMO cause (n, %)  Lung transplantation  ARDS  Other pulmonary | 17 (34.0)  18 (36.0)  15 (30.0) | 14 (28.0)  18 (36.0)  18 (36.0) | 13 (25.5)  16 (31.4)  22 (43.1) | 4 (8.0)  23 (46.0)  23 (46.0) | 48 (23.9)  75 (37.3)  78 (38.8) | 0.588 |
| ECMO VA mode (n, %) | 9 (18.0) | 6 (12.0) | 9 (17.6) | 8 (16.0) | 32 (16.1) | 0.967 |
| ECMO settings  Blood flow rate (L/min)  FsO_2_ (%) | 3.1±0.8  94.6±12.2 | 3.0±0.7  93.8±12.3 | 3.2±0.9  91.1±13.6 | 3.0±0.8  85.4±17.1 | 3.1±0.8  91.2±14.3 | 0.952  0.001 |
| ECMO duration (days)*^*^* | 8 (4-19) | 10 (5-16) | 8 (4-17) | 9 (4-23) | 9 (4-18) | 0.735 |
| Mechanical ventilation (n, %) | 50 (100.0) | 50 (100.0) | 51 (100.0) | 50 (100.0) | 201 (100.0) | >0.999 |
| WBC (x10^3^/μL) | 11.2±5.8 | 14.2±7.3 | 13.7±8.3 | 13.9±8.8 | 13.3±7.7 | 0.13 |
| Hemoglobin (g/dL) | 10.5±1.8 | 11.1±2.2 | 10.1±1.8 | 10.4±2.7 | 10.5±2.2 | 0.318 |
| BUN (mg/dL) | 28.6±21.3 | 21.7±13.6 | 25.3±13.9 | 28.5±23.9 | 26.0±18.8 | 0.766 |
| Creatinine (mg/dL) | 1.0±1.0 | 0.8±0.4 | 1.0±0.6 | 1.0±0.7 | 1.0±0.7 | 0.993 |
| eGFR (mL/min/1.73m^2^) | 115.7±67.1 | 114.0±56.0 | 103.2±61.2 | 99.2±53.2 | 108.0±59.5 | 0.12 |
| Albumin (g/dL) | 2.5±0.6 | 2.7±0.5 | 2.5±0.6 | 2.5±0.7 | 2.6±0.6 | 0.488 |
| Bilirubin (mg/dL) | 1.7±2.4 | 1.5±1.9 | 2.3±2.2 | 1.6±1.8 | 1.8±2.1 | 0.868 |
| CRP (mg/dL) *^*^* | 69.7  (11.1-237.9) | 89.3  (39.0-165.9) | 129.2  (26.0-205.7) | 110.5  (25.9-156.1) | 105.3  (23.8-182.6) | 0.514 |
| APACHE II | 23.1±9.0 | 24.2±8.9 | 21.6±8.1 | 24.5±9.3 | 23.3±8.8 | 0.762 |
| Incident AKI (%) | 18 (36.0) | 10 (20.0) | 26 (51.0) | 27 (54.0) | 81 (40.3) | 0.006 |
| CRRT treatment (%) | 5 (10.0) | 3 (6.0) | 12 (23.5) | 15 (30.0) | 35 (17.4) | 0.001 |

^*^Data are expressed as median and interquartile ranges and compared by Jonckheere-Terpstra test.

^†^Fluid balance during entire ECMO treatment

*Abbreviations* : CVD, cardiovascular disease; CFB, cumulative fluid balance for 3 days from ECMO initiation; DM, diabetes mellitus; ECMO, extracorporeal membrane oxygenation; VA, veno-arterial access; CVD, cardiovascular disease; ARDS, acute respiratory distress syndrome; FsO_2_, fraction of sweep gas inlet oxygen supplied to ECMO; WBC, white blood cell; BUN, blood urea nitrogen; eGFR, estimated glomerular filtration rate; CRP, C-reactive protein; APACHE II, acute physiology and chronic health evaluation II; AKI, acute kidney injury; CRRT, continuous renal replacement therapy.

**Table S3.** Cox regression analyses for 90 day mortality with ENCOURAGE and PRESERVE scores

| **Variables** | **Cardiovascular disease^*^** | | **Non-cardiovascular disease**^†^ | |
| --- | --- | --- | --- | --- |
|  | **HR (95% CI)** | **P** | **HR (95% CI)** | **P** |
| CFB**^‡^** + ENCOURAGE | 1.48 (1.15-1.90) | 0.003 | - | - |
| CFB**^‡^** + PRESERVE | - | - | 1.23 (1.04-1.44) | 0.013 |

**^*^**Further adjusted Charlson comorbidity index in the CVD group.

^†^Further adjusted sex and Charlson comorbidity index in patients undergone VV-ECMO due to respiratory disease.

**^‡^**Data were log transformed.

*Abbreviations* : CFB, cumulative fluid balance; ENCOURAGE, prediction of cardiogenic shock outcome for AMI patients salvaged by VA-ECMO; PRESERVE, predicting death for severe ARDS on VV-ECMO; AMI, acute myocardial infarction; VA, veno-arterial; ECMO, extracorporeal membrane oxygenation; ARDS, acute respiratory distress syndrome; VV, veno-venous.
