# Supplementary figures and images for: Permissive fluid volume in adult patients undergoing extracorporeal membrane oxygenation treatment
Source: Crit Care. 2018 Oct 27;22:270. doi: 10.1186/s13054-018-2211-x (PMC6203979; doi:10.1186/s13054-018-2211-x)

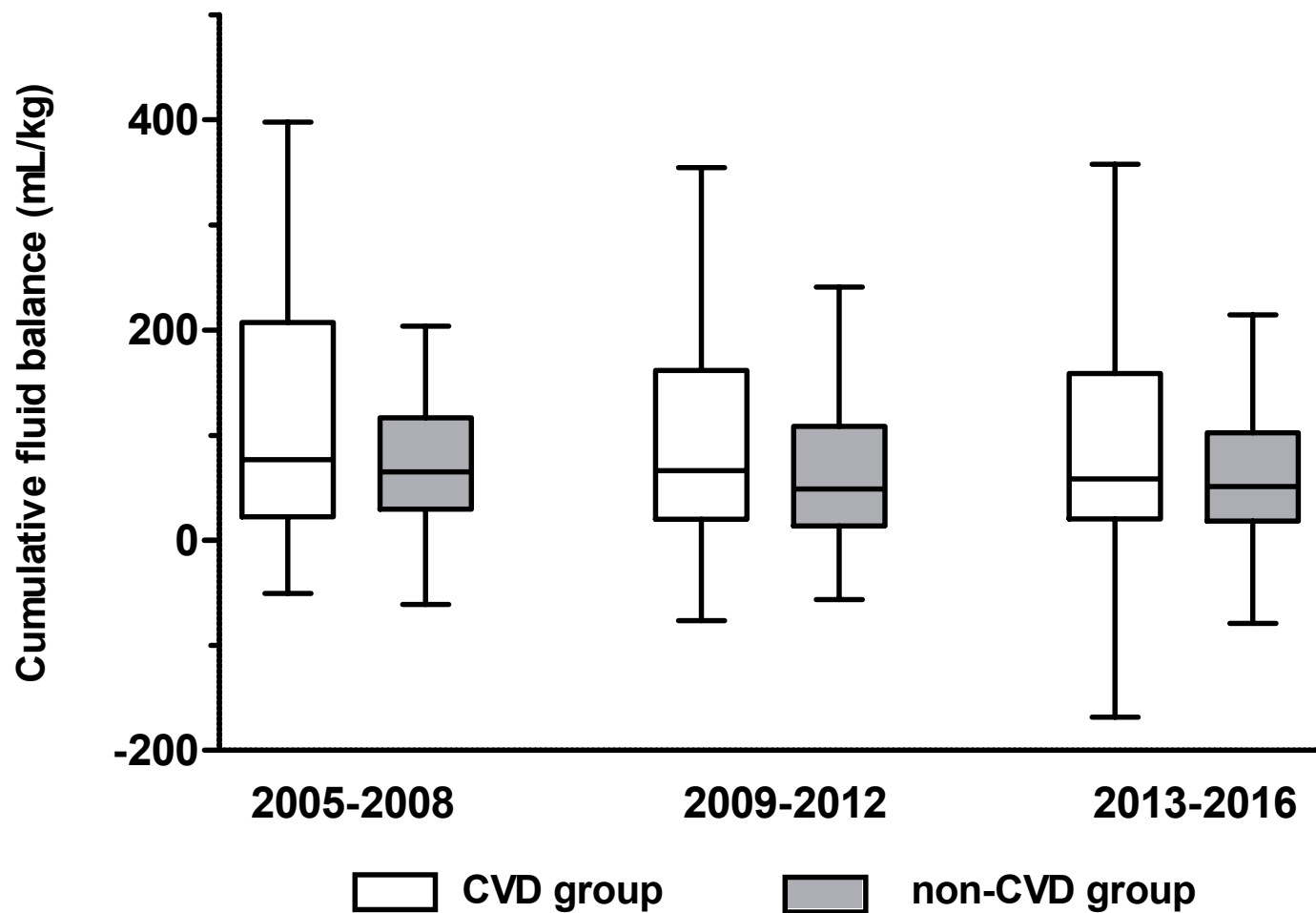

**Figure S1.** Cumulative fluid balance according to ECMO treatment vintage.

Supplement: Supplementary file 2 — Figure S1. Cumulative fluid balance according to ECMO treatment vintage. (PDF 31 kb) [file 13054_2018_2211_MOESM2_ESM.pdf]
